# The chloroplast genome of *Spiraea thunbergii* (Rosaceae)

**DOI:** 10.1080/23802359.2022.2135406

**Published:** 2022-10-27

**Authors:** Wanying Shen, Ji Lin, Haifeng Lin

**Affiliations:** College of Information Science and Technology, Nanjing Forestry University, Nanjing, Jiangsu, China

**Keywords:** *Spiraea thunbergii*, chloroplast genome, phylogenetic analysis

## Abstract

*Spiraea thunbergii* (*S. thunbergia*) is a very common ornamental shrub in the garden, with important horticultural and economic value. In this study, we assembled the complete chloroplast (cp) genome of *S. thunbergia* into a typical quadripartite structure. The genome size and GC content of the *S. thunbergia* cp genome are 155,922 bp and 36.76%, respectively. It contains 84 protein-coding genes, 37 tRNA genes, and 8 rRNA genes. The phylogenetic tree supported that *S. thunbergii* is closely related to *Spiraea mongolica* in the Rosaceae family. The study will provide significant genomic resource for elucidating the phylogenetic relationship of *Spiraea*.

*Spiraea thunbergii* Carl Peter Thunberg, 1784 (Figure 1), also known as Thunberg spirea, baby’s breath spirea, or breath of spring spirea, belongs to the Rosaceae family. Spiraea plants are known for their beautiful white flowers that in 3- to 5-flowered clusters, so they are very common ornamental shrubs in gardens and have important horticultural and economic value (Yu et al. 2018). *S. thunbergia* is native to eastern China and is now widely cultivated as an ornamental plant in China, Korea, and Japan. Recently, some chloroplast (cp) genomes of the genus *Spiraea* have been reported (Huo et al. 2019, Qin et al. 2022), but few studies have been reported on *S. thunbergia*. In this study, we assembled and characterized the complete cp genome of *S. thunbergia*, which will enrich the genetic information of *S. thunbergia* and contribute to the species identification of the *Spiraea* genus.

**Figure 1.**
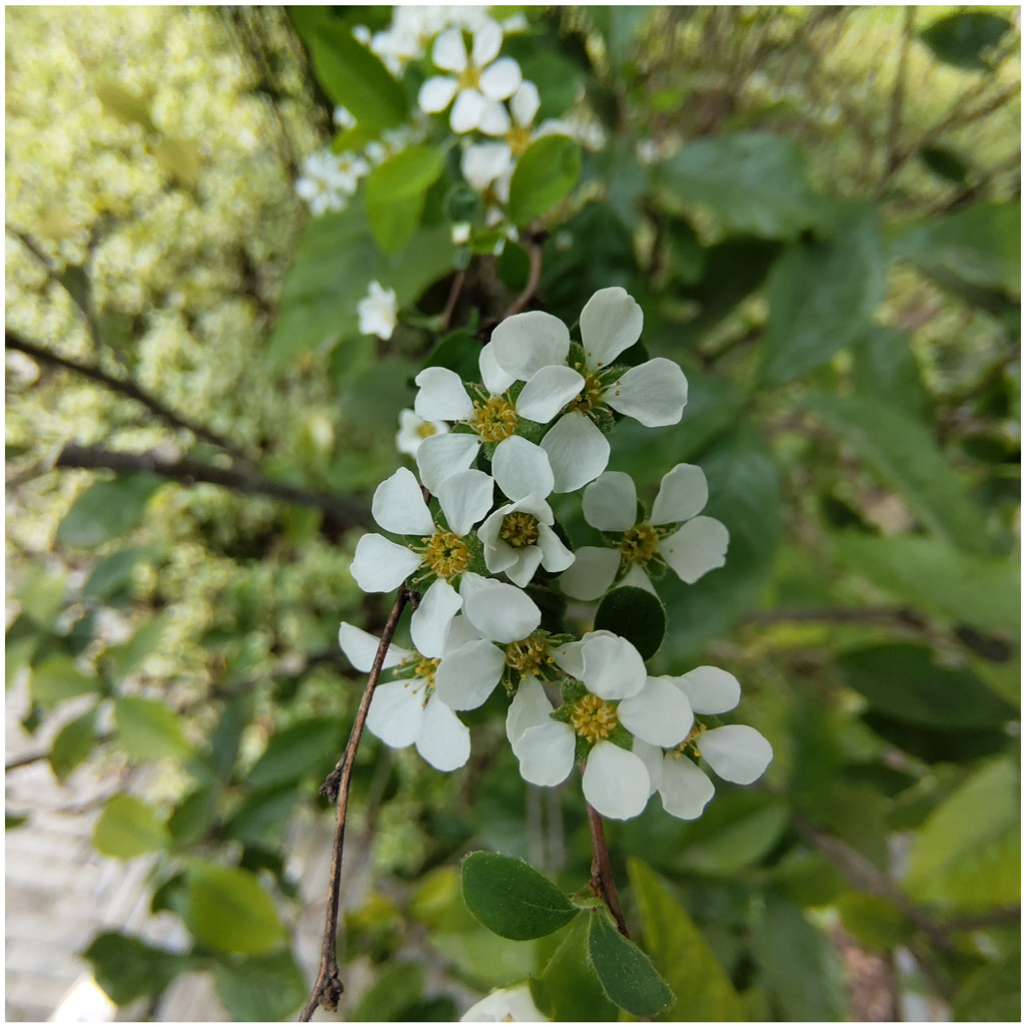
The reference image of *Spiraea thunbergii.*

The experimental sample of *S. thunbergia* was collected from Nanjing, China (32°4'32.6′'N, 118°48'45′'E) and deposited at Nanjing Forestry University (www.njfu.edu.cn, accession number: NFU20220311ZXJ, Haifeng Lin: haifeng.lin@njfu.edu.cn). The genomic DNA was extracted from the fresh leaves using the modified CTAB method (Doyle and Doyle 1987, Bi et al. 2022). The total DNA was then fragmented to construct an Illumina paired-end library and sequenced using the Illumina NovaSeq 6000 platform. The raw sequencing data were filtered and trimmed by the fastp v0.23 program (Chen et al. 2018, Ma et al. 2022), and then fed into GetOrganelle v1.7.5 for assembly (Jin et al. 2020). As shown in Figure S1, the cp genome of *S. thunbergia* was assembled into a typical quadripartite structure (Wick et al. 2015). The assembled cp genome of *S. thunbergia* was then annotated with CpGAVAS2 (Shi et al. 2019) using the cp genome of *Spiraea mongolica* (GenBank accession: NC_051992.1.1) as the reference. Finally, the complete cp genome of *S. thunbergia* was submitted to GenBank (accession no. NC_064734.1).

The complete cp genome of *S. thunbergii* is 155,922 bp in length with a typical quadripartite structure (Figure 2), composing of a large single copy (LSC) region of 84,360 bp, a small single copy (SSC) region of 18,880 bp, and a pair of inverted repeats (IRs) regions of 26,341 bp. The overall GC content of *S. thunbergii* cp genome is 36.76%, which is higher than that of LSC (34.61%) and SSC (30.35%), but lower than IRs (42.5%). The *S. thunbergii* cp genome encodes 129 genes, including 84 protein-coding genes, 37 tRNA genes, and 8 rRNA genes. A total of 22 genes were found to contain one intron, including 14 protein-coding genes (*rps*16, *atp*F, *rpo*C1, *rps1*2 × 2, *clp*P, *pet*B, *pet*D, *rpl*16, *rpl*2 × 2, *ndh*B × 2, and *ndh*A), and 8 tRNA genes (*trn*K-UUU, *trn*G-GCC, *trn*L-UAA, *trn*V-UAC, *trn*I-GAU × 2, and *trn*A-UGC × 2), and only one gene (*ycf*3) contains two introns (Figure S2). Additionally, *rps*12 is a trans-spliced gene with 5′ end located in the LSC region and the duplicated 3′ end in the IR regions (Figure S3). Additionally, we also detected the tandem repeats and microsatellite sequences in the *S. thunbergia* cp genome, which were incorporated into the output circular map for visualization (Figure 2).

**Figure 2.**
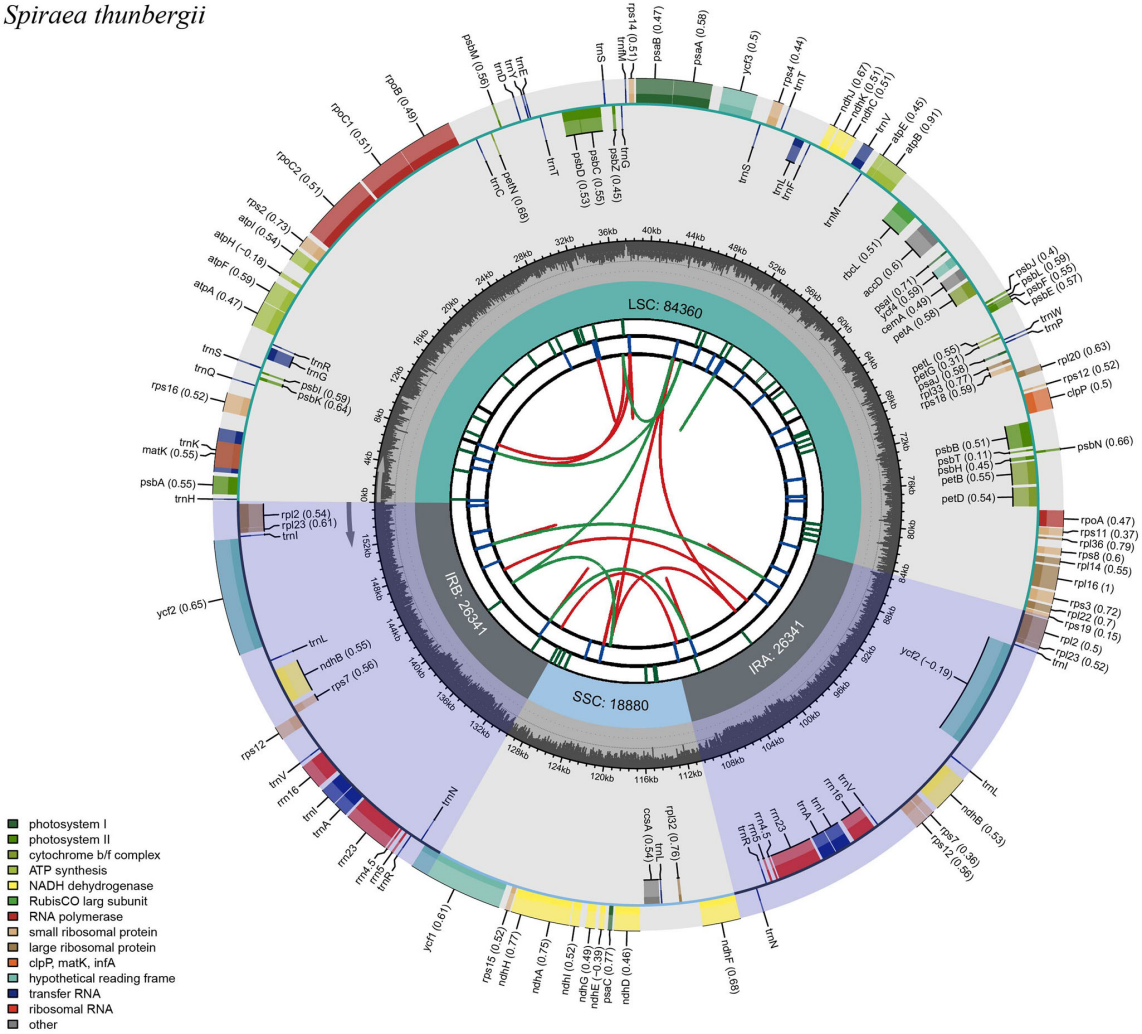
The genome map of *Spiraea thunbergii* chloroplast genome. From the center going outward, the first circle shows the forward and reverse repeats connected with red and green arcs, respectively. The second and third circles show the tandem repeats and microsatellite sequences marked with short bars, respectively. The outer circle shows the gene structure on the chloroplast genome. The genes were colored based on their functional categories, which are shown at the left corner.

**Figure 3.**
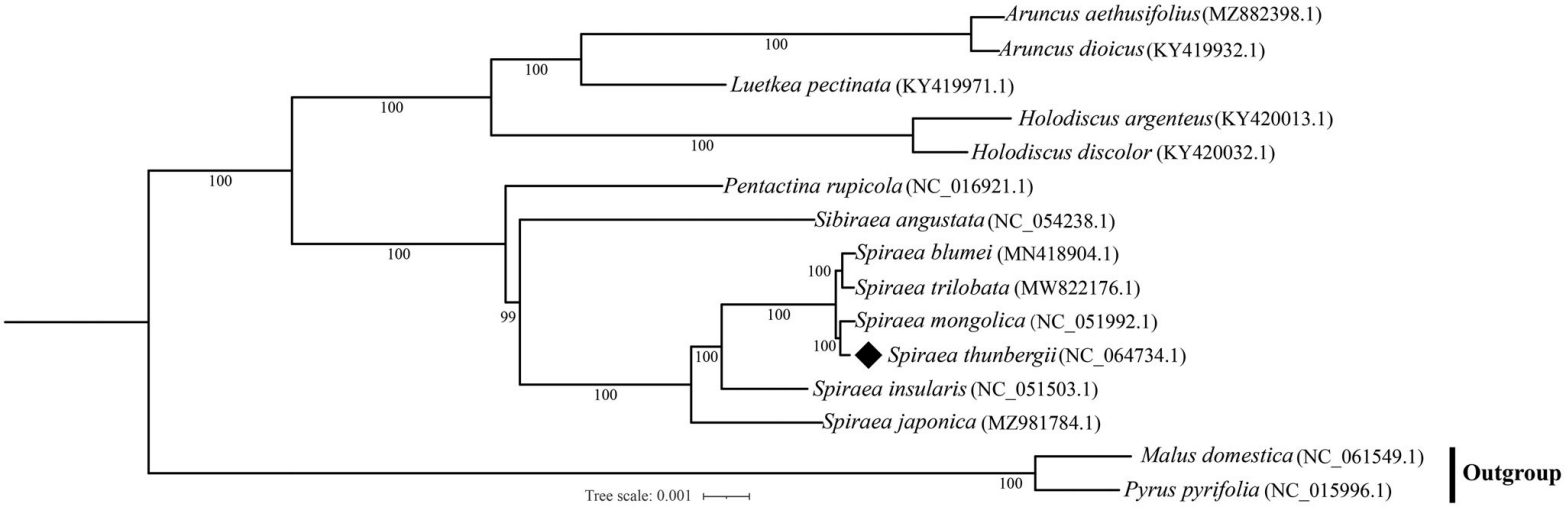
Maximum Likelihood tree based on 15 complete cp genome sequences. The numbers on branches are bootstrap support values from 1000 replicates. Accession numbers are listed right to their scientific names.

In order to determine the phylogenetic position of *S. thunbergii*, 14 other cp genomes from Amygdaloideae were obtained from NCBI to reconstruct the Maximum Likelihood (ML) tree. *Malus domestica* and *Pyrus pyrifolia* were used as the outgroup species. These complete cp genomes were first aligned using MAFFT v7.49 (Katoh and Standley 2013), and the gaps in the alignment were trimmed using trimAl v1.4 (Capella-Gutierrez et al. 2009). The ML phylogenetic tree was constructed using IQTREE v2.2 with 1000 bootstrap replicates (Minh et al. 2020). The best evolutionary model was chosen as ‘UNREST + FO + R3’ according to the Bayesian Information Criterion (BIC) scores generated from IQ-TREE. The phylogenetic result suggested that *S. thunbergii* is the sister of *Spiraea mongolica*, and they are evolutionarily close to *Sibiraea angustata* and *Pentactina rupicola* in the family Rosaceae (Figure 3). This study of *S. thunbergii* cp genome will provide more genomic resources for the identification and application of *Spiraea*.

## Supplementary Material

Supplemental Material

Supplemental Material

Supplemental Material

## Data Availability

The genome sequence data that support the findings of this study are openly available in GenBank of NCBI under the accession no. NC_064734.1. The associated BioProject, SRA, and BioSample numbers are PRJNA835237, SRR19090745, and SAMN28097313, respectively.

## References

[CIT0001] Bi C, Qu Y, Hou J, Wu K, Ye N, Yin T. 2022. Deciphering the multi-chromosomal mitochondrial genome of Populus simonii. Front Plant Sci. 13(:914635. 35783945 10.3389/fpls.2022.914635 PMC9240471

[CIT0002] Capella-Gutierrez S, Silla-Martinez JM, Gabaldon T. 2009. trimAl: a tool for automated alignment trimming in large-scale phylogenetic analyses. Bioinformatics. 25(15):1972–1973. 19505945 10.1093/bioinformatics/btp348 PMC2712344

[CIT0003] Chen S, Zhou Y, Chen Y, Gu J. 2018. fastp: an ultra-fast all-in-one FASTQ preprocessor. Bioinformatics. 34(17):i884–i890. 30423086 10.1093/bioinformatics/bty560 PMC6129281

[CIT0004] Doyle JJ, Doyle JL. 1987. A rapid DNA isolation procedure for small quantities of fresh leaf tissue. Phytochem Bull. 19:11–15.

[CIT0005] Huo Y, Yan M, Zhao X, Zhu Z, Yuan Z. 2019. The complete chloroplast genome sequence of *Spiraea blumei* G. Don (Rosaceae). Mitochondrial DNA Part B. 4(2):3671–3672. 33366136 10.1080/23802359.2019.1678434 PMC7707486

[CIT0006] Jin JJ, Yu WB, Yang JB, Song Y, dePamphilis CW, Yi TS, Li DZ. 2020. GetOrganelle: a fast and versatile toolkit for accurate de novo assembly of organelle genomes. Genome Biol. 21(1):241. 32912315 10.1186/s13059-020-02154-5 PMC7488116

[CIT0007] Katoh K, Standley DM. 2013. MAFFT multiple sequence alignment software version 7: improvements in performance and usability. Mol Biol Evol. 30(4):772–780. 23329690 10.1093/molbev/mst010 PMC3603318

[CIT0008] Ma Q, Wang Y, Li S, Wen J, Zhu L, Yan K, Du Y, Ren J, Li S, Chen Z, et al. 2022. Assembly and comparative analysis of the first complete mitochondrial genome of *Acer truncatum* Bunge: a woody oil-tree species producing nervonic acid. BMC Plant Biol. 22(1):29. 10.1186/s12870-021-03416-5 PMC8756732 35026989

[CIT0009] Minh BQ, Schmidt HA, Chernomor O, Schrempf D, Woodhams MD, von Haeseler A, Lanfear R. 2020. IQ-TREE 2: new models and efficient methods for phylogenetic inference in the genomic era. Mol Biol Evol. 37(5):1530–1534. 32011700 10.1093/molbev/msaa015 PMC7182206

[CIT0010] Qin H, Zhu X, Zhang X, Zhang X, Zhang L. 2022. Characterization of the complete plastome of *Spiraea trilobata* (Rosaceae), a perennial shrub. Mitochondrial DNA Part B. 7(1):249–250. 35087944 10.1080/23802359.2021.2018948 PMC8788365

[CIT0011] Shi L, Chen H, Jiang M, Wang L, Wu X, Huang L, Liu C. 2019. CPGAVAS2, an integrated plastome sequence annotator and analyzer. Nucleic Acids Res. 47(W1):W65–W73. 31066451 10.1093/nar/gkz345 PMC6602467

[CIT0012] Wick RR, Schultz MB, Zobel J, Holt KE. 2015. Bandage: interactive visualization of de novo genome assemblies. Bioinformatics. 31(20):3350–3352. 26099265 10.1093/bioinformatics/btv383 PMC4595904

[CIT0013] Yu SX, Gadagkar SR, Potter D, Xu DX, Zhang M, Li ZY. 2018. Phylogeny of Spiraea (Rosaceae) based on plastid and nuclear molecular data: Implications for morphological character evolution and systematics. Perspect Plant Ecol Evol Syst. 34:109–119.

