# Supplementary figures and images for: The chloroplast genome of Spiraea thunbergii (Rosaceae)
Source: Mitochondrial DNA B Resour. 2022 Oct 27;7(10):1879–81. doi: 10.1080/23802359.2022.2135406 (PMC9621243; doi:10.1080/23802359.2022.2135406)

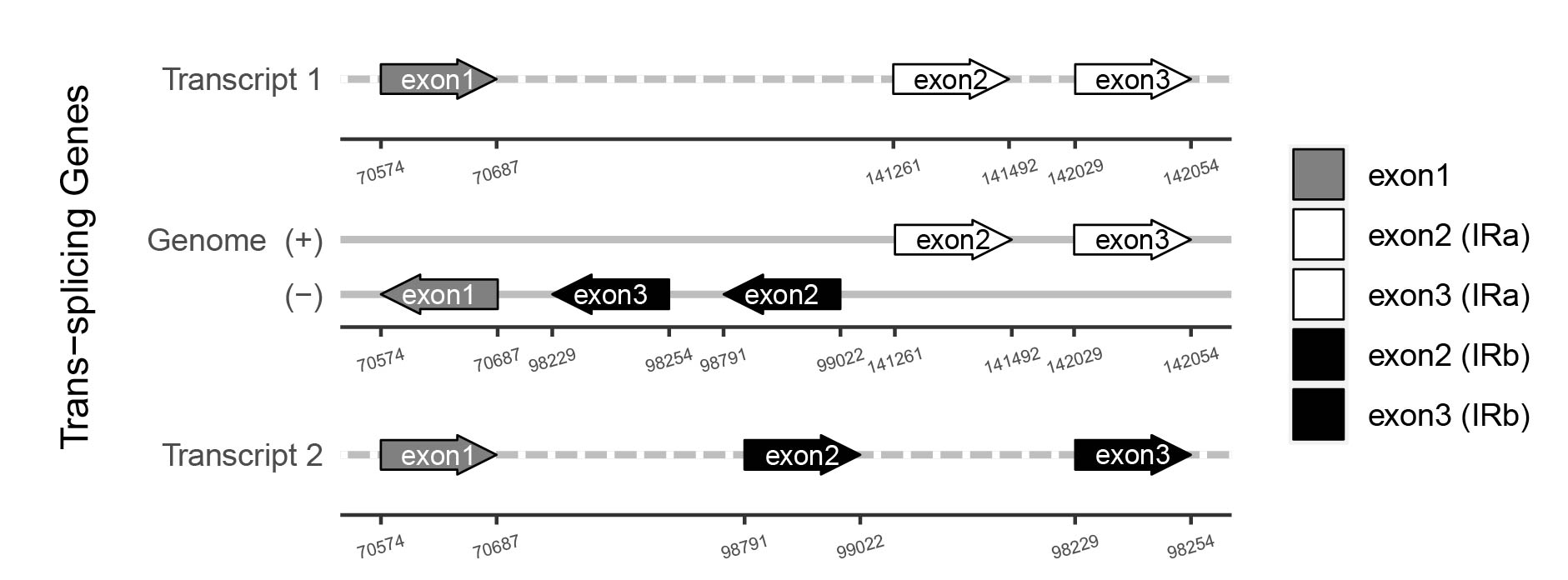

Supplement: Supplemental Material [file TMDN_A_2135406_SM7205.jpg]

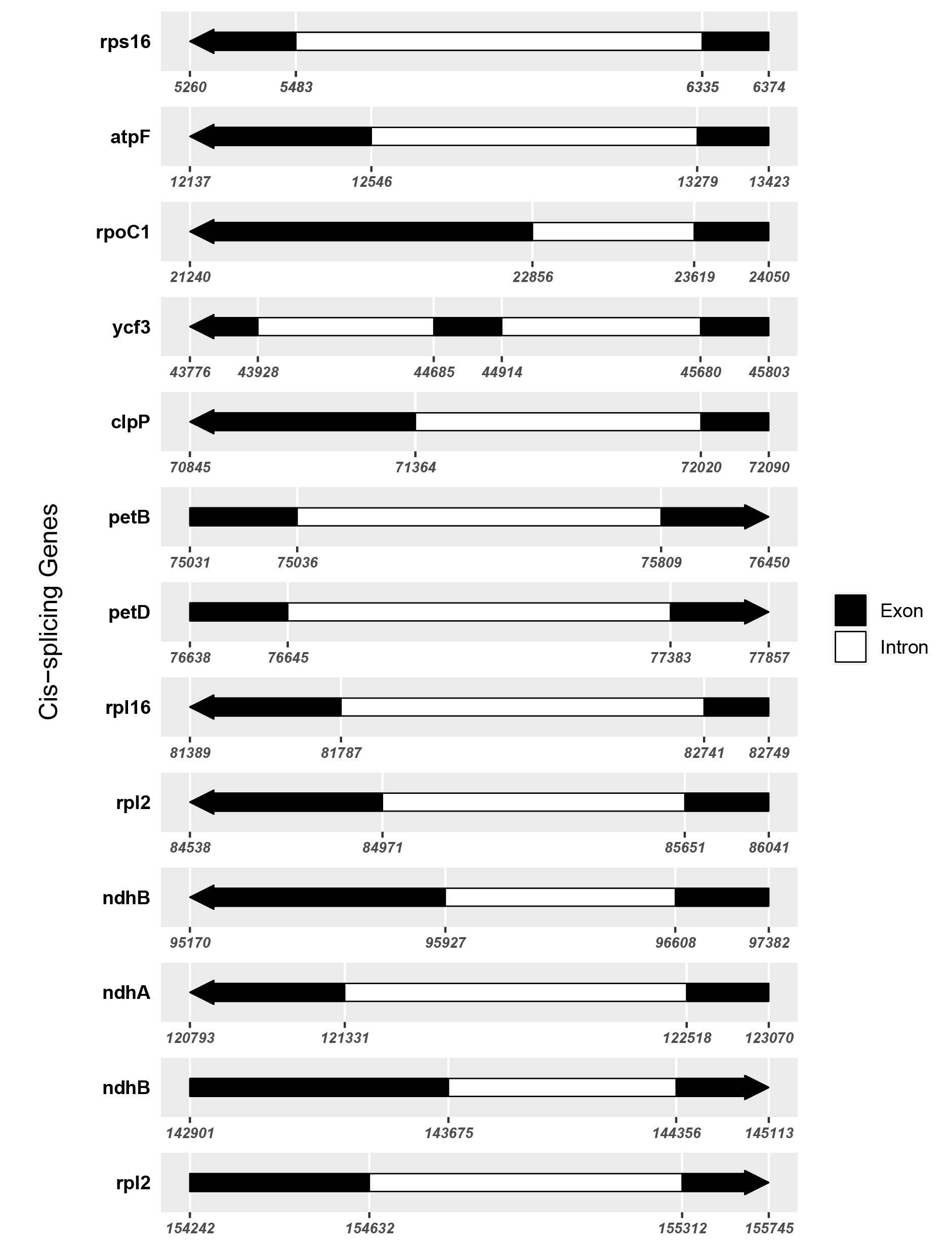

Supplement: Supplemental Material [file TMDN_A_2135406_SM7202.jpg]

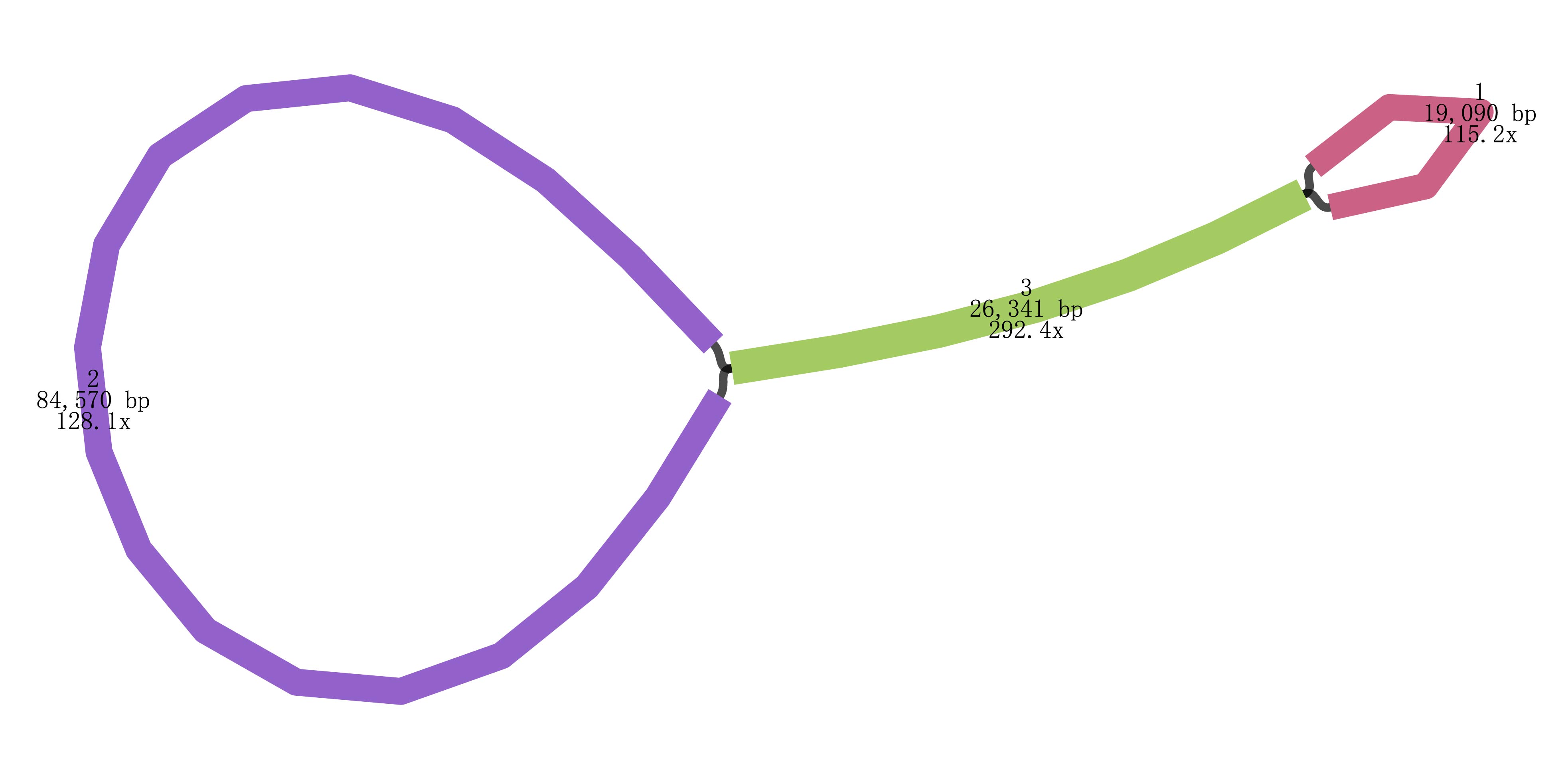

Supplement: Supplemental Material [file TMDN_A_2135406_SM7159.jpg]
